# Associations of the COVID-19 pandemic with the reported incidence of important endemic infectious disease agents and syndromes in Pakistan

**DOI:** 10.1186/s12879-022-07869-3

**Published:** 2022-11-26

**Authors:** Bayan Missaghi, Muhammad Wasif Malik, Waseem Shaukat, Muazam Abbas Ranjha, Aamer Ikram, Herman W. Barkema

**Affiliations:** 1 Department of Medicine, Cumming School of Medicine, University of Calgary and Alberta Health Services, Office 3683, 3500 – 26 Ave NE, Calgary, AB T1Y 6J4 Canada; 2 Infection Prevention and Control, Calgary Zone, Alberta Health Services, Calgary, AB Canada; 3 Field Epidemiology & Disease Surveillance Division, National Institute of Health (NIH), Islamabad, Pakistan; 4 Department of Production Animal Health, Faculty of Veterinary Medicine, University of Calgary, 3330 Hospital Drive NW, HSC 2561, Calgary, AB Canada; 5 Executive Office, National Institute of Health (NIH), Islamabad, Pakistan; 6 Department of Community Health Sciences, Cumming School of Medicine, University of Calgary, Office HS2521, 3330 Hospital Dr. NW, Calgary, AB T2N 4N1 Canada; 7 One Health at UCalgary, University of Calgary, Calgary, AB Canada

**Keywords:** COVID-19, SARS-CoV-2, Infectious disease outbreaks, Incidence

## Abstract

**Background:**

Persons in Pakistan have suffered from various infectious diseases over the years, each impacted by various factors including climate change, seasonality, geopolitics, and resource availability. The COVID-19 pandemic is another complicating factor, with changes in the reported incidence of endemic infectious diseases and related syndromes under surveillance.

**Methods:**

We assessed the monthly incidence of eight important infectious diseases/syndromes: acute upper respiratory infection (AURI), viral hepatitis, malaria, pneumonia, diarrhea, typhoid fever, measles, and neonatal tetanus (NNT), before and after the onset of the COVID-19 pandemic. Administrative health data of monthly reported cases of these diseases/syndromes from all five provinces/regions of Pakistan for a 3-year interval (March 2018–February 2021) were analyzed using an interrupted time series approach. Reported monthly incidence for each infectious disease agent or syndrome and COVID-19 were subjected to time series visualization. Spearman’s rank correlation coefficient between each infectious disease/syndrome and COVID-19 was calculated and median case numbers of each disease before and after the onset of the COVID-19 pandemic were compared using a Wilcoxon signed-rank test. Subsequently, a generalized linear negative binomial regression model was developed to determine the association between reported cases of each disease and COVID-19.

**Results:**

In late February 2020, concurrent with the start of COVID-19, in all provinces, there were decreases in the reported incidence of the following diseases: AURI, pneumonia, hepatitis, diarrhea, typhoid, and measles. In contrast, the incidence of COVID was negatively associated with the reported incidence of NNT only in Punjab and Sindh, but not in Khyber Pakhtunkhwa (KPK), Balochistan, or Azad Jammu & Kashmir (AJK) & Gilgit Baltistan (GB). Similarly, COVID-19 was associated with a lowered incidence of malaria in Punjab, Sindh, and AJK & GB, but not in KPK and Balochistan.

**Conclusions:**

COVID-19 was associated with a decreased reported incidence of most infectious diseases/syndromes studied in most provinces of Pakistan. However, exceptions included NNT in KPK, Balochistan and AJK & GB, and malaria in KPK and Balochistan. This general trend was attributed to a combination of resource diversion, misdiagnosis, misclassification, misinformation, and seasonal patterns of each disease.

**Supplementary Information:**

The online version contains supplementary material available at 10.1186/s12879-022-07869-3.

## Background

Over the years, persons in Pakistan have experienced a wide variety of infectious diseases such as Dengue fever, Crimean-Congo Hemorrhagic fever (CCHF), viral hepatitis, measles, and polio [1]. The diversity of healthcare challenges over time has been exacerbated by many outbreaks of endemic transmissible pathogens, with devastating consequences both from a morbidity and mortality perspective [2], as well as from the standpoint of healthcare costs [3–5]. Unfortunately, as is the case with many developing nations with struggling healthcare sectors, Pakistan has also been quite vulnerable to effects of the novel coronavirus 2019 (COVID-19) pandemic [6], caused by the severe acute respiratory syndrome coronavirus 2 (SARS-CoV-2). Government estimates state that, as of December 1, 2021, the current pandemic had infected more than 1.25 million Pakistanis and taken more than 28,000 lives [7]. Globally, as of the same date, the pandemic had involved 222 countries, infected > 250,000,000 people, and taken more than 5 million lives [8].

The incidence of ongoing non-COVID-19 infectious diseases endemic in Pakistan is influenced by a complex set of evolving circumstances, involving factors such as climate change, global warming, seasonality, loss of biodiversity, changes in vector geography, and many other ecological determinants [1]. Moreover, Pakistan’s attempts at control of various outbreaks have suffered from geopolitical factors, misinformation, and a shortage of resources, as exemplified by the ongoing polio outbreak in the tribal-controlled regions of the country [9, 10]. In this context, isolating impacts of any one infectious disease outbreak on another is neither simple nor straightforward.

Nevertheless, despite the inherent complexity of the causes and interplay between determinants, it has been postulated that there have been notable impacts of COVID-19 on the incidence and/or prevalence of several infectious diseases in Pakistan, including arboviral diseases, influenza A virus subtype H1N1, Dengue fever, Crimean-Congo hemorrhagic fever, tuberculosis, malaria, measles, typhoid fever, etc. [11–16]. Some of these impacts have been attributed to under-reporting and a diversion of resources from the diagnosis and management of ongoing endemic pathogens such as measles and polio to the diagnosis and surveillance of COVID-19, whereas others have been proposed to be due to possible exacerbation of these ongoing other outbreaks by COVID-19 (in a synergistic fashion) and/or over-reporting thereof due to an overlap in symptoms. Early on in the pandemic, there were also efforts made by the National Command and Operations Centre (NCOC) of the Government of Pakistan to develop standard operating procedures that were meant to be strictly followed in public areas, including guidelines on physical distancing, wearing masks, maintaining hand hygiene, and implementing lockdowns [17]. Consequently, it is likely that these efforts had an inadvertent impact on lowering the incidence of other transmissible infections as well, particularly those spread through a respiratory route. Regardless of the specific etiologies, several Pakistani scientists and clinicians have observed anecdotal alterations in the incidence rates of various infectious diseases currently under surveillance throughout the country. However, the directionality of these changes and their degree have not been comprehensively described. Therefore, the objectives were to assess the impact of the COVID-19 pandemic on the reported incidence of endemic infectious diseases/syndromes in Pakistan, and to discuss potential underlying reasons for any such impact.

## Methods

We received permission from the Ministry of National Health Services Regulations and Coordination (MoNHSR&C) of the Government of Pakistan to obtain monthly incident infectious disease data for the following pathogens and infectious disease syndromes: acute upper respiratory infection, viral hepatitis (primarily hepatitis A and E), malaria (undifferentiated species), pneumonia (undifferentiated), diarrhea (undifferentiated), typhoid fever, measles, neonatal tetanus and COVID-19 for 3 years (March 2018–February 2021) for all five provinces/regions of Pakistan—namely Punjab, Sindh, Khyber Pakhtunkhwa (KPK), Balochistan, and Azad Jammu & Kashmir (AJK) & Gilgit Baltistan (GB). This interval was from 24 months before to 12 months after the onset of COVID-19 in Pakistan that occurred at the end of February 2020. Data regarding the monthly incidence of COVID-19 were collected from the time of the first case in late February 2020 through February 2021.

Case definitions of the eight diseases discussed in the present study were adopted from the Pakistan List of Priority Diseases for Implementation of the Integrated Disease Surveillance Response System (IDSR), as follows:

1. Acute upper respiratory infection (AURI)—Acute upper respiratory infection with cough, rhinorrhea or sore throat in the absence of clinical or x-ray evidence of pneumonic involvement.
2. Pneumonia—Cough or difficulty breathing with fever, chills, chest pain and clinical or x-ray evidence of pneumonic consolidation.
3. Viral hepatitis (A & E)—Acute onset of jaundice < 1 month duration and severe illness (dark urine, fatigue, nausea, vomiting, and abdominal pain) with absence of any known precipitating factors (other than contaminated food or water).
4. Diarrhea (Non-Cholera)—An illness characterized by ≥ 3 loose, non-bloody stools over a 24-h interval, with or without dehydration.
5. Typhoid fever—Acute febrile illness, fever of ≥ 38 °C for ≥ 3 days with abdominal discomfort, fatigue and diarrhea/constipation.
6. Measles—Any person in whom a clinician suspects measles infection OR any person with fever AND any 2 of the following signs/symptoms: maculopapular rash (non-vesicular), cough, coryza, or conjunctivitis.
7. Neonatal tetanus (NNT)—Any neonate between 3 and 28 days of age with, in the absence of a more likely diagnosis, an acute illness with muscle spasms or hypertonia and a clinical diagnosis of tetanus by a health care provider, OR death from neonatal tetanus listed on the death certificate as the cause of death or a significant condition contributing to death.
8. Malaria (undifferentiated species)—Any person with fever or history of fever within the past 48 h (with or without other symptoms such as nausea, vomiting and diarrhea, headache, back pain, chills, or myalgia) in whom other obvious causes of fever have been excluded.
9. COVID-19—Acute onset of any 3 or more of the following signs or symptoms: fever, dry cough, general weakness/fatigue, headache, myalgia, sore throat, coryza, dyspnea, anorexia/nausea/vomiting and diarrhea, particularly in the context of having exposure to a known positive case or residing in a high-transmission area within 14 d prior to the onset of symptoms.

The entire population of Pakistan, including persons of all age groups, sexes, educational status, socio-economic status, and residence, was included in this study. Non-residents of Pakistan were excluded. A standardized tool used by the MoNHSR&C for the collection of aggregated data for their Demographic Health Information System (DHIS) was adopted, specifically targeting eight priority infectious diseases/syndromes in the country in addition to COVID-19. Of note, the DHIS works at the national level in Pakistan under the administrative control of the MoNHSR&C, Government of Pakistan. All districts across Pakistan report data monthly to the DHIS for various diseases. Specifically, data flowed from 17,003 data reporting sites, including health facilities, up to the district level, and then it was subsequently combined at a provincial level and transmitted to the National Health Ministry database.

### Data Analyses

Statistical analyses were performed using Stata/SE 17.0 for Mac (StataCorp. 2021. Stata Statistical Software: Release 17. College Station, TX: StataCorp LLC). The reported incidence of all eight infectious diseases before and after the onset of the COVID-19 pandemic in Pakistan was analyzed using an interrupted time series approach. Reported monthly incidence of each infectious disease pathogen or syndrome was assessed for normality using histograms and the Shapiro–Wilk test. These data were non-normally distributed, and, therefore, case counts were reported as medians along with interquartile range (IQR). Time series line plots were created for each disease and province, respectively, representing monthly case numbers along with COVID-19 case numbers to visually compare the cases in each of the 3 years in the dataset, including 2 years before (March 2018–February 2020) and 1 year after the onset of COVID-19 (March 2020–February 2021). Subsequently, the Spearman’s correlation coefficient (*ρ*) between each separate infectious disease pathogen or syndrome and COVID-19 was calculated using a two-tailed α < 0.05 defined as meeting significance. To determine whether the number of monthly cases for any disease differed between the pre-COVID and COVID period, a Wilcoxon signed-rank test was used to compare monthly averages of the pre-COVID period with the COVID period, considering the seasonal variations between months in a year. Boxplots were created to visually assess the distribution of cases between pre-COVID and COVID periods for each of the infectious diseases/syndromes in each province/region.

Thereafter, in view of overdispersion in these count data, a generalized linear negative binomial regression model with log link was developed, which allowed adequate handling of over-dispersed count data to determine associations between COVID-19 and each of the infectious disease pathogens or syndromes separately for each province. To account for seasonal trends in the data, monthly indicator variables were included in the final models. The final fitted model that we report in this study, separately for each disease and province, was this equation:

$$\begin{aligned} \log \left( {E\left( {Disease} \right)} \right) = & \beta_{0} + \beta_{1} \cdot \left( {COVID \,cases \,in \,Thousands} \right) \\ & + \mathop \sum \limits_{i = 1}^{12} \beta_{i} \cdot I_{{Month_{i} }} , \\ \end{aligned}$$

where E(Disease) is the expected monthly number of the cases of disease for which the model is fitted, β_0_ is the log of the expected count of disease cases when COVID-19 cases were 0 for the month of January as the base, β_1_ is the difference in the log of the expected count of disease cases with every 1000 increase in COVID-19 cases at any given month, and β_i_ is the difference in the log of expected count of disease cases between January (as a base) and the respective month.

In view of autocorrelation in this time series data, we applied heteroskedasticity and autocorrelation-consistent (HAC) variance estimator using Newey–West as a kernel to compute the HAC standard errors for point estimates from the models. Finally, model residuals for autocorrelation were validated using Breusch–Godfrey and Cumby–Huizinga tests [18]. Model fit was assessed by visualization of residuals and approximation of fitted values. Finally, we exponentiated the model coefficients to report incidence rate ratios (IRRs) along with 95% confidence intervals (CIs) to reflect associations of COVID-19 cases with reporting of various infectious diseases.

Due to logistical challenges in consistent data collection throughout the study period, there were intervals for specific diseases in certain provinces with missing data, including data for all diseases in KPK province for October 2019 and for malaria in Sindh province for August 2018 to March 2019. All missing points pertained to the pre-pandemic period and were not imputed.

## Results

**Fig. 1:**
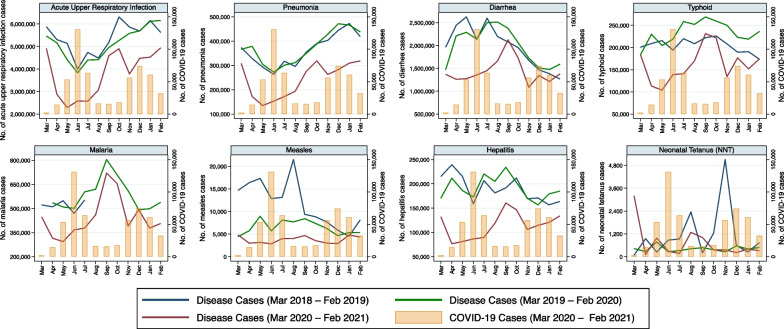
Monthly number of reported cases of various infectious diseases or syndromes in Pakistan from March 2018 to February 2021 and cases of COVID-19 from March 2020 to February 2021

**Fig. 2:**
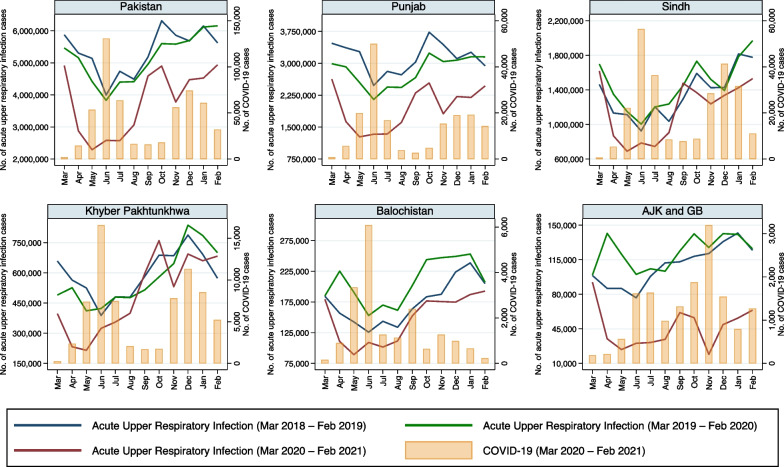
Monthly number of reported cases of acute upper respiratory infection (AURI) and COVID-19 in five provinces/regions of Pakistan from March 2018 to February 2021

**Fig. 3:**
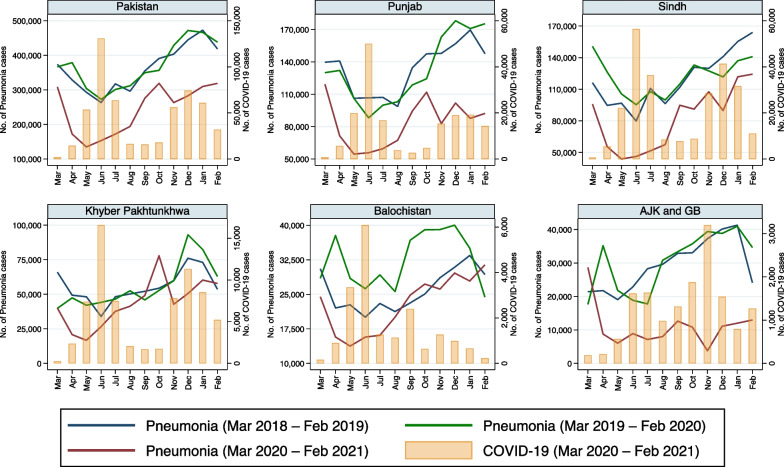
Monthly reported cases of pneumonia and COVID-19 in five provinces/regions of Pakistan from March 2018 to February 2021

**Fig. 4:**
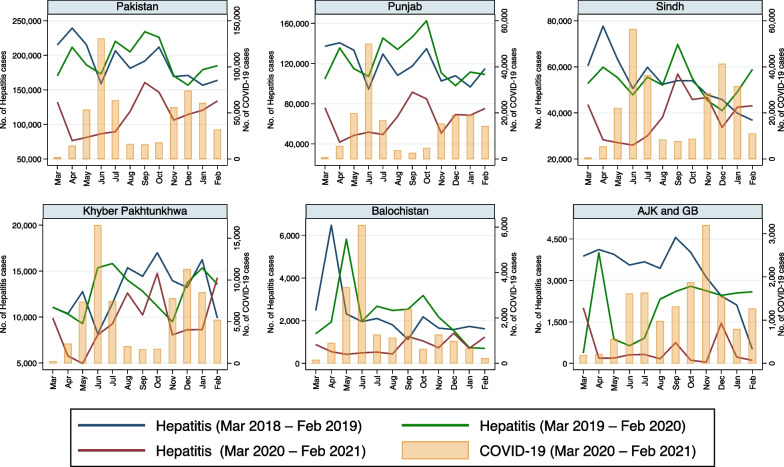
Monthly reported cases of hepatitis and COVID-19 in five provinces/regions of Pakistan from March 2018 to February 2021

**Fig. 5:**
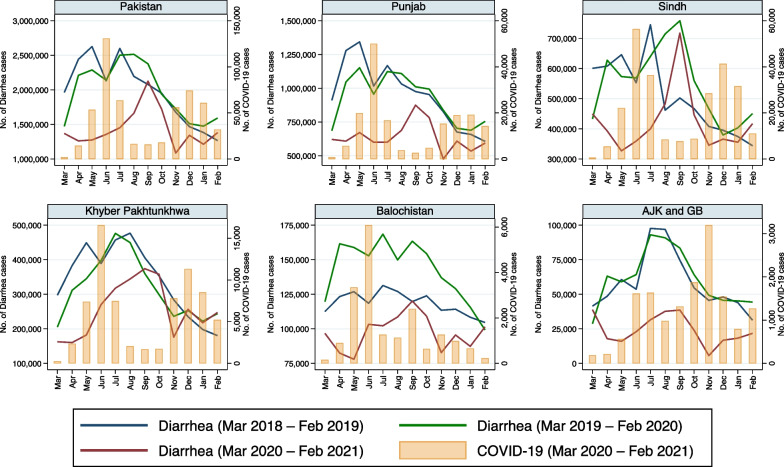
Monthly reported cases of diarrhea and COVID-19 in five provinces/regions of Pakistan from March 2018 to February 2021

**Fig. 6:**
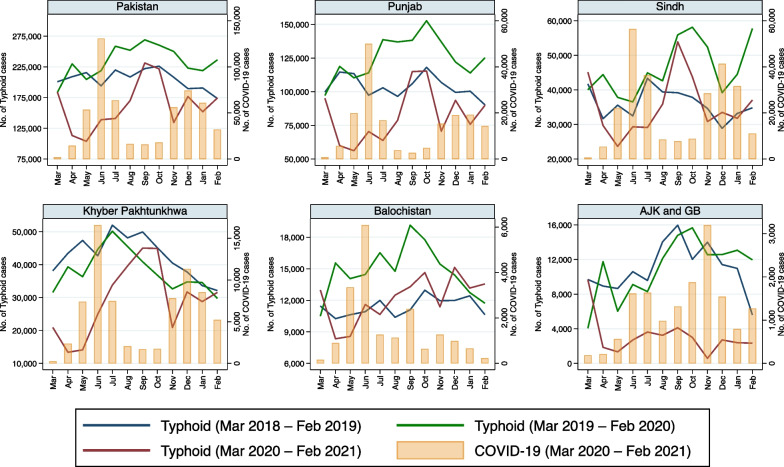
Monthly reported cases of typhoid and COVID-19 in five provinces/regions of Pakistan from March 2018 to February 2021

**Fig. 7:**
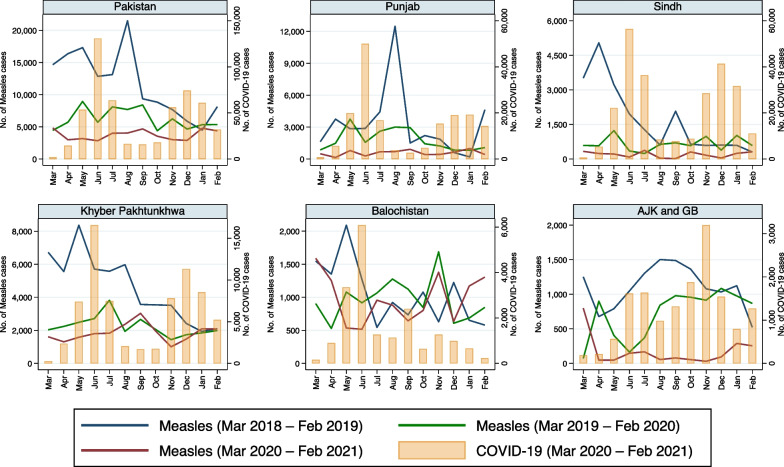
Monthly reported cases of measles and COVID-19 in five provinces/regions of Pakistan from March 2018 to February 2021

**Fig. 8:**
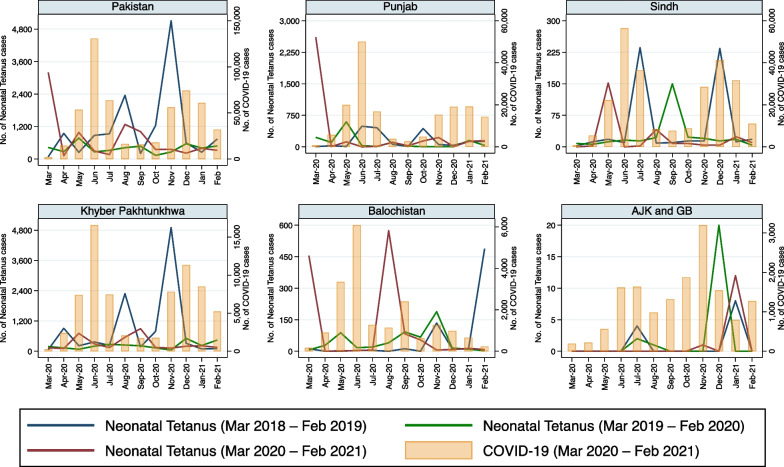
Monthly reported cases of NNT and COVID-19 in five provinces/regions of Pakistan from March 2018 to February 2021

**Fig. 9:**
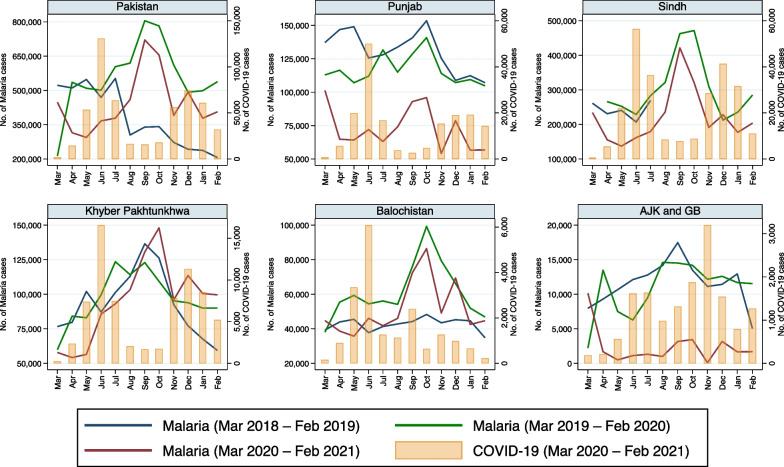
Monthly reported cases of malaria and COVID-19 in five provinces/regions of Pakistan from March 2018 to February 2021

Reported monthly incidence of each of the eight infectious disease pathogens/syndromes decreased in all provinces of Pakistan with the advent of COVID-19 in late February 2020 (Fig. 1). Moreover, the incidence of all these diseases remained lower in 2020–2021 than in the pre-pandemic period, especially when COVID-19 cases peaked (Figs. 1, 2, 3, 4, 5, 6, 7, 8, 9). Reported incidence of COVID-19 and incidence of pneumonia, hepatitis and diarrhea were negatively correlated in all five provinces (p < 0.05), whereas a negative correlation was observed for measles and typhoid in all provinces except Balochistan (Table 1). Notably, a negative correlation between AURI and COVID-19 occurred in all provinces except for Sindh and KPK. Similarly, the reported incidence of malaria was negatively correlated in all provinces except KPK and Balochistan. However, the incidence of NNT was only negatively correlated in Sindh province. Median monthly reported cases of all diseases along with interquartile range (Q1–Q3) in the pre-pandemic and pandemic periods in various provinces/regions are presented in Table 2 and visualized in Additional file 1: Fig. S1.

**Table 1:** Spearman correlation of COVID-19 cases with various infectious diseases/syndromes in various provinces of Pakistan

	Punjab		Sindh		Khyber Pakhtunkhwa		Balochistan		AJK & GB^a^	
	r_s_	p value	r_s_	p value	r_s_	p value	r_s_	p value	r_s_	p value
Acute upper respiratory infection	− 0.752	< 0.001	− 0.266	0.118	− 0.227	0.189	− 0.510	0.002	− 0.799	< 0.001
Pneumonia	− 0.721	< 0.001	− 0.529	0.001	− 0.339	0.046	− 0.445	0.006	− 0.772	< 0.001
Hepatitis	− 0.819	< 0.001	− 0.592	< 0.001	− 0.562	< 0.001	− 0.735	< 0.001	− 0.742	< 0.001
Diarrhea	− 0.701	< 0.001	− 0.514	0.001	− 0.375	0.026	− 0.680	< 0.001	− 0.759	< 0.001
Typhoid	− 0.679	< 0.001	− 0.363	0.029	− 0.562	< 0.001	− 0.138	0.426	− 0.761	< 0.001
Malaria	− 0.820	< 0.001	− 0.554	0.002	0.029	0.867	− 0.045	0.794	− 0.758	< 0.001
Measles	− 0.666	< 0.001	− 0.742	< 0.001	− 0.558	< 0.001	− 0.118	0.494	− 0.728	< 0.001
Neonatal tetanus	− 0.012	0.943	− 0.394	0.017	− 0.085	0.626	− 0.077	0.654	− 0.036	0.834

^a^Azad Jammu & Kashmir & Gilgit Baltistan

**Table 2:** Monthly number of cases of various infectious diseases/syndromes in various provinces of Pakistan before and after the onset of COVID-19 pandemic expressed as median (Q1–Q3)

	Monthly cases Pre-COVID period (March 2018–February 2020)	Monthly cases COVID period (March 2020–February 2021)	p value
Acute upper respiratory infection			
Punjab	3,069,346 (2,735,657–3,218,780)	2,006,049 (1,474,394–2,385,241)	< 0.001
Sindh	1,391,410 (1,169,758–1,620,949)	1,287,438 (826,934–1,451,322)	0.003
Khyber Pakhtunkhwa	549,015 (478,170–665,718)	465,123 (339,966–671,673)	0.005
Balochistan	187,799 (161,662–215,482)	163,602 (109,895–177,966)	< 0.001
AJK & GB^a^	116,112 (102,663–127,583)	42,116 (30,764–58,881)	< 0.001
Pneumonia			
Punjab	135,383 (104,878–158,489)	85,240 (63,440–98,109)	< 0.001
Sindh	121,051 (105,231–132,694)	90,485 (53,528–101,750)	< 0.001
Khyber Pakhtunkhwa	51,385 (47,393–59,902)	42,099 (32,135–54,206)	0.002
Balochistan	29,717 (25,860–32,946)	24,649 (15,922–27,566)	0.002
AJK & GB	29,795 (21,942–36,362)	9871 (7582–12,321)	0.001
Hepatitis			
Punjab	121,129 (105,419–134,753)	67,946 (50,033–75,602)	< 0.001
Sindh	53,351 (47,236–58,453)	40,325 (29,190–44,709)	0.001
Khyber Pakhtunkhwa	11,772 (11,061–13,648)	8943 (8060–11,423)	0.005
Balochistan	1951 (1699–2538)	720 (512–1140)	0.001
AJK & GB	2431 (2212–3144)	210 (140–537)	< 0.001
Diarrhea			
Punjab	980,016 (743,933–1,108,243)	608,436 (596,977–679,985)	< 0.001
Sindh	538,899 (417,356–613,838)	396,529 (357,571–448,058)	0.009
Khyber Pakhtunkhwa	347,301 (244,320–397,148)	251,935 (179,117–330,954)	0.024
Balochistan	137,060 (118,872–142,008)	99,075 (85,077–105,934)	< 0.001
AJK & GB	57,419 (45,728–69,461)	22,335 (17,320–34,625)	0.001
Typhoid			
Punjab	114,277 (107,429–121,366)	77,258 (67,090–94,348)	< 0.001
Sindh	40,952 (37,368–45,210)	32,629 (29,515–40,423)	0.016
Khyber Pakhtunkhwa	41,403 (34,867–45,410)	30,171 (20,901–36,690)	0.003
Balochistan	12,797 (12,461–13,982)	12,732 (11,021–13,422)	0.266
AJK & GB	11,171 (8862–13,178)	2710 (2097–3436)	0.001
Malaria			
Punjab	124,713 (114,852–130,712)	68,394 (59,933–85,812)	< 0.001
Sindh	247,570 (232,257–262,272)	197,501 (169,485–235,045)	0.125
Khyber Pakhtunkhwa	92,535 (78,840–112,447)	97,759 (71,764–108,433)	0.700
Balochistan	49,232 (47,222–57,941)	45,421 (42,173–59,299)	0.970
AJK & GB	11,525 (9145–13,089)	1681 (1069–3175)	0.001
Measles			
Punjab	2223 (1396–3072)	570 (424–746)	0.002
Sindh	796 (617–1718)	227 (64–309)	< 0.001
Khyber Pakhtunkhwa	3900 (2080–4378)	1814 (1525–2085)	0.007
Balochistan	951 (860–1129)	918 (639–1279)	0.970
AJK & GB	917 (679–1110)	86 (51–211)	0.001
Neonatal tetanus			
Punjab	100 (47–226)	113 (9–143)	0.733
Sindh	15 (12–49)	6 (2–17)	0.258
Khyber Pakhtunkhwa	274 (160–520)	176 (140–423)	0.413
Balochistan	17 (11–48)	8 (5–68)	0.910
AJK & GB	0 (0–2)	0 (0–0)	0.750

^a^Azad Jammu & Kashmir & Gilgit Baltistan

### Respiratory infections

Median number of reported AURI cases decreased in the pandemic period compared to the pre-pandemic period in all provinces/regions (Table 2). As the cases of COVID-19 began in late February 2020 and peaked in June 2020 in Punjab and in AJK & GB, the reported number of AURI cases decreased compared to the preceding years’ cases (Fig. 2). The lowest incidence of AURI occurred between May and July 2020 and approached the preceding years’ reported incidence from August onwards until November 2020 when another dip concurrent with the second wave of COVID-19 in these provinces. Similar trends were observed for Sindh, KPK and Balochistan in which a reduced incidence of AURI occurred during the first wave of COVID-19, though the second wave did not seem to be associated with as much a decline in incidence of AURI. The regression model revealed that when seasonal trends are controlled for, an increase in COVID-19 cases was associated with a reduction in the IRR of AURI in all provinces/regions, particularly in AJK & GB (Table 3).

**Table 3:** Overall interrupted time series analysis using generalized linear negative binomial regression models adjusted for seasonality for various infectious diseases/syndromes as dependent variables and COVID-19 cases in thousands as an independent variable in various provinces/regions of Pakistan

	Punjab		Sindh		Khyber Pakhtunkhwa		Balochistan		AJK & GB^b^	
	IRR^a^ (95% CI)	p value	IRR (95% CI)	p value	IRR (95% CI)	p value	IRR (95% CI)	p value	IRR (95% CI)	p value
Acute upper respiratory infection	0.981 (0.979, 0.983)	< 0.001	0.993 (0.992, 0.994)	< 0.001	0.976 (0.970, 0.980)	< 0.001	0.906 (0.878, 0.935)	< 0.001	0.527 (0.509, 0.546)	< 0.001
Pneumonia	0.979 (0.975, 0.983)	< 0.001	0.987 (0.985, 0.988)	< 0.001	0.962 (0.955, 0.968)	< 0.001	0.895 (0.864, 0.928)	< 0.001	0.474 (0.462, 0.487)	< 0.001
Hepatitis	0.976 (0.972, 0.980)	< 0.001	0.989 (0.987, 0.991)	< 0.001	0.959 (0.952, 0.967)	< 0.001	0.695 (0.636, 0.760)	< 0.001	0.250 (0.217, 0.289)	< 0.001
Diarrhea	0.984 (0.983, 0.986)	< 0.001	0.992 (0.990, 0.993)	< 0.001	0.975 (0.968, 0.982)	< 0.001	0.903 (0.874, 0.934)	< 0.001	0.521 (0.507, 0.536)	< 0.001
Typhoid	0.985 (0.981, 0.988)	< 0.001	0.994 (0.991, 0.996)	< 0.001	0.959 (0.952, 0.965)	< 0.001	0.956 (0.922, 0.990)	0.012	0.386 (0.365, 0.407)	< 0.001
Malaria	0.979 (0.975, 0.983)	< 0.001	0.991 (0.990, 0.992)	< 0.001	1.000 (0.991, 1.010)	0.946	0.981 (0.940, 1.023)	0.369	0.268 (0.242, 0.298)	< 0.001
Measles	0.955 (0.947, 0.963)	< 0.001	0.951 (0.945, 0.958)	< 0.001	0.941 (0.923, 0.959)	< 0.001	0.875 (0.854, 0.896)	< 0.001	0.278 (0.248, 0.311)	< 0.001
Neonatal tetanus	0.943 (0.903, 0.985)	0.008	0.953 (0.917, 0.990)	0.014	0.980 (0.933, 1.030)	0.427	0.790 (0.597, 1.046)	0.099	0.851 (0.555, 1.304)	0.457

^a^Incidence rate ratio

^b^Azad Jammu & Kashmir & Gilgit Baltistan

A similar pattern was observed with pneumonia (Table 2). Reported pneumonia cases decreased with the advent of increasing reported COVID-19 cases in 2020–2021 compared to the reported pneumonia cases in the same months in the preceding years in Punjab and Sindh (Fig. 3). A similar pattern was observed in KPK and Balochistan, but only for the first wave of the pandemic. In AJK & GB, there was a sharp decrease in reported pneumonia cases with the onset of the pandemic and the lowest numbers of reported cases were observed in November 2020 when COVID-19 cases peaked in these regions (Fig. 3). The regression model demonstrated that the reported pneumonia cases decreased in all provinces/regions, with an increase of reported COVID-19 cases, when seasonality was included in the model (Table 3).

### Food and waterborne diseases

Reported incidence of viral hepatitis in all provinces/regions decreased at the start of the pandemic (Table 2, Fig. 4) and was consistently lower in the pandemic period compared to the preceding years in Punjab, especially during both waves of COVID-19. Viral hepatitis incidence decreased compared to preceding years in Sindh, but only during the first wave of COVID-19. Similar trends were observed in other provinces. The regression model adjusted for seasonality revealed that an increase of reported COVID-19 cases was associated with reduced IRRs of viral hepatitis in all provinces/regions (Table 3).

A similar pattern was observed with diarrhea in all five provinces/regions (Table 2). Our regression model revealed that the increase of COVID-19 cases was associated with lowering the incidence of reported cases of diarrhea by 1.6% in Punjab, 0.8% in Sindh, 2.5% in KPK, 9.7% in Balochistan, and 47.9% in AJK & GB (Table 3). In addition, median reported cases of diarrhea during the pandemic period (2020–2021) were lower compared to the corresponding pre-pandemic period (2018–2020) in all provinces/regions (Table 2). The incidence of diarrhea cases did not increase from March to July in 2020 as it did for the 2 preceding years in Punjab (Fig. 5). As the incidence of COVID-19 declined after July 2020, the incidence of diarrhea approached the preceding years’ levels during the fall of 2020, and, with the second wave of COVID-19, another decline from the previous years’ levels was observed in Punjab. However, in Sindh, KPK, Balochistan and AJK & GB, the reported number of diarrhea cases was lower than the previous years’ only during the first wave of COVID-19 but not during the second wave (Fig. 5).

Reported incidence of typhoid was also negatively associated with increasing cases of COVID-19 in our regression model that depicted a significant association between typhoid and COVID-19 in all provinces/regions (Table 3). Median reported cases of typhoid fever were higher during the corresponding pre-pandemic compared to the pandemic period in Punjab, Sindh, KPK, and AJK & GB, but not Balochistan (Table 2). There was a sharp reduction in the number of cases of typhoid in Punjab, compared to previous years, as the incidence of COVID-19 increased between March and July 2020, as well as in November 2020. A similar reduction in cases of typhoid occurred in this province as did in KPK—with a bimodal reduction of cases during the two waves of the COVID-19 pandemic in 2020. Of note, Sindh province had a reduction in typhoid incidence during the first wave of the pandemic only, whereas aside from a small reduction of typhoid cases in April and May 2020 during the first wave of the pandemic in Balochistan, there was no notable change in reported incidence in that province. In contrast, in AJK & GB, typhoid incidence dropped precipitously after the onset of the pandemic and remained significantly lower than that seen in previous years throughout the study period (Fig. 6).

### Vaccine preventable diseases

Median recorded cases of measles differed in the pre-pandemic vs. the pandemic period all provinces/regions but Balochistan (Table 2, Fig. 7). However, when seasonality was incorporated, reported cases of measles were negatively associated with pandemic cases in all provinces/regions (Table 3). Reductions in measles incidence during the first wave of the pandemic occurred in all five provinces/regions, whereas reductions in measles incidence during the second wave primarily occurred in Punjab, Sindh, and AJK & GB and not in KPK or Balochistan (Fig. 7).

Incidence of NNT did not differ in the pre-pandemic vs. the pandemic period in any province: (Table 2). However, the regression model incorporating seasonality revealed that reported cases of NNT were negatively associated with pandemic cases in Punjab and Sindh, but not in KPK, Balochistan and AJK & GB (Table 3). The incidence of NNT was lower in Punjab in the first wave of the pandemic but not during the second wave, whereas the reverse was true for Sindh (Fig. 8). Furthermore, there was no difference in the reported incidence of NNT in KPK, Balochistan and AJK & GB between the pre-pandemic and the pandemic periods (Fig. 8).

### Vector-borne diseases

Reported incidence of malaria differed in the pre-pandemic vs the pandemic period only in Punjab and AJK & GB. Interestingly, our regression model demonstrated that reported cases of malaria were negatively associated with pandemic cases in Punjab, Sindh, and AJK & GB, in contrast with KPK and Balochistan, where no significant difference was detected (Table 3). The incidence of malaria was lower during both the first and second waves of the pandemic in Punjab, Sindh and AJK & GB. Conversely, there was no difference in malaria case numbers between the pre-pandemic and pandemic periods in Balochistan, although a possible trend towards a decreased number of cases occurred during the first wave of the COVID-19 pandemic in KPK (Fig. 9).

## Discussion

The reported incidence of several infectious disease pathogens/syndromes decreased after the first case of COVID-19 was reported in Pakistan in late February 2020. The decrease occurred for all eight infectious disease pathogens/syndromes in most of the provinces/regions included in this study: AURI, pneumonia, viral hepatitis, diarrhea, typhoid fever, measles, malaria and NNT. The only exceptions were malaria in KPK and Balochistan, as well as NNT in KPK, Balochistan and AJK & GB, which demonstrated no difference in IRRs between the pre-pandemic and pandemic periods—after seasonality was accounted for. A potential reason that these two particular infectious diseases did not demonstrate a significant difference in all five provinces/regions is that the risk factors, clinical manifestations, and diagnosis of these two diseases are, generally, different enough from that of COVID-19 so as not to cause confusion and lead to frequent misclassification or misdiagnosis [19, 20]. Presumably, there was sufficient priority and interest in the control and prevention of malaria and NNT in Pakistan, to ensure that resources were not diverted from them to combat the COVID-19 pandemic [21, 22] in every province. Moreover, in respect to NNT specifically, case numbers in every province were relatively limited at the outset (i.e., in the tens to hundreds), likely as a result of vaccination efforts over the years. As an illustration, there were no cases of NNT in AJK & GB during the study period.

The incidence of several of the diseases such as malaria are very affected by seasonal fluctuations, likely related to the life cycle of its mosquito vectors [23] and other weather-related factors. It is therefore important to include seasonality in the model when analyzing the association between reported COVID-19 cases and incidence of these diseases. This is illustrated by the fact that when using the Wilcoxon signed-rank test (which does not take the varying trend of COVID-19 cases into account), the reported incidence of the 8 diseases was associated with the reported incidence of COVID-19 in less provinces/regions than in the regression models that included seasonality.

Monthly incidence of most of the infectious disease pathogens/syndromes (AURI, pneumonia, viral hepatitis, diarrhea, typhoid fever, and measles) decreased with the advent of COVID-19 in late February 2020 overall in the entire country (Fig. 1) as well as in all five provinces/regions separately (Figs. 2, 3, 4, 5, 6, 7, 8, 9). A potential explanation is that after the first COVID-19 cases were reported in late February 2020, precautionary measures such as the closure of schools, work from home policies, public health restrictions and lockdowns were implemented [24], which adversely affected surveillance and data reporting of these diseases. In addition, the outpatient treatment departments (OPDs) of all major hospitals across the country were closed for patients during the initial few weeks of the pandemic [24, 25] leading to a substantial effect on the inflow of patients to the hospitals (potential data points for surveillance) for reasons other than COVID-19. This explains a sudden drop in reported cases of almost all major ongoing infectious diseases in all provinces. Subsequently, different provinces adopted different strategies to re-open under their respective administrative controls. With the onset of pandemic, a sudden increase in the “fear factor” associated with forced isolation and perceived stigma around a novel disease, largely spread by hyper-reactive and sensational media reports [26], also negatively affected the public’s willingness to seek medical attention through qualified doctors and hospitals, replacing this instead with a surge in self-prescription and home remedies for various health problems [27, 28]. This trend is likely one of the main reasons for a sudden decline of reported incidence of various diseases with the escalating pandemic in many developing countries such as Pakistan. Alternatively, or perhaps additionally, several of these infectious disease pathogens/syndromes have presentations which overlap with those of COVID-19, particularly considering the purely clinically based case definitions used by the Pakistan IDSR, and may have been mistakenly diagnosed as such [16].

Some interesting findings became clear when modelling these infectious disease pathogens/syndromes. It is not surprising to see an inverse relationship between the number of COVID-19 cases and the number of AURI cases (Fig. 2), given the overlap in symptoms between these 2 conditions. The diagnosis of AURI is made clinically and is essentially defined as an upper respiratory tract infection (URTI), whereas upper respiratory symptoms, including sore throat, cough and rhinorrhea, are well documented symptoms of COVID-19 [29]. As such, given the overlap in symptoms and lack of clear diagnostic testing for AURI, it is highly likely that COVID-19 could have been mistaken for AURI and vice versa, especially in a developing setting with less access to diagnostic testing. A similar argument could be made for pneumonia (Fig. 3), given the overlap in symptoms between these two conditions. Pneumonia due to COVID-19 is well described [30] and, thus, there is ample opportunity for misclassification between COVID-19 pneumonia and pneumonia due to other pathogens, particularly in a developing setting with less access to distinct and differential diagnostic testing.

There was a clear inverse relationship between COVID-19 cases and the number of reported cases of viral hepatitis (Fig. 4), believed to be mainly food and waterborne hepatitis due to hepatitis A and E. As the number of cases of COVID-19 in Pakistan increased, peaked in June 2020 and then declined, the number of reported cases of viral hepatitis steadily decreased, plateaued and then began to increase and approach the expected incidence again by September 2020 in most province/regions. We believe that the inverse relationship in this case may be primarily due to a diversion of resources, including diagnostic testing capacity, from viral hepatitis towards COVID-19 testing and management, although it may also be due to an overlap in symptoms during the pre-icteric phase of viral hepatitis, resulting in misdiagnosis and misclassification.

Similarly, the reported incidence of diarrhea in Pakistan declined with the onset of COVID-19 cases in late February 2020 (Fig. 5), perhaps because diarrhea is a common manifestation of COVID-19 [16, 29]. As expected, as COVID-19 case numbers decreased in July and August 2020, reported cases of diarrheal illness continued to increase and peaked in September 2020 (Fig. 6). Notably, typhoid fever is mainly transmitted through exposure to contaminated food and water, the latter of which is more likely to be consumed in larger quantities during the hot summer months. Regardless, there was a clear negative impact of the onset of COVID-19 cases in late February 2020 on the reported incidence of typhoid fever, and a similar phenomenon occurred in October through December 2020. The general inverse correlation between typhoid fever and COVID-19 was likely due to both a diversion of resources from the former to the latter as well as an overlap in symptoms such as fever, weakness, diarrhea and cough—resulting in misdiagnosis and misclassification [31].

The incidence of reported measles cases also decreased after the advent of COVID-19 cases in late February 2020, whereas reductions in COVID-19 cases since that time were associated with increased reporting of measles cases (Fig. 7). This was attributed to a diversion of resources during the COVID-19 pandemic previously put towards measles diagnostic testing, tracing and management. Of interest, the impact of the pandemic on the reporting of measles cases was significant during the first wave of the pandemic, where all provinces had a notable decline in case numbers, whereas in Balochistan and AJK & GB the same reduction in incidence did not occur during the second wave. This may have been due to some level of adjustment and improvement in measles diagnostic testing and tracing that were in place by this point in time in those provinces/regions.

The NNT case numbers were low at the outset (Fig. 8), and apparently least affected by the pandemic of all diseases/syndromes that we explored, demonstrating a small reduction during the first wave of the pandemic in Punjab and during the second wave of the pandemic in Sindh, but not otherwise. Malaria case numbers were impacted by the onset of the pandemic in some provinces/regions more than others, with a notable decrease in case numbers in Punjab, Sindh and AJK & GB (Fig. 9). As alluded to, this infectious disease was the most impacted by seasonal variations, as demonstrated by the difference between our Wilcoxon signed-rank test and our negative binomial regression model.

Despite a growing body of literature attempting to speculate, define and describe the impact of the COVID-19 pandemic on other ongoing infectious diseases outbreaks in many developing settings, to our knowledge our study is the first original research paper to do this using infectious diseases surveillance data from the MoNHSR&C. This had several advantages: we were able to utilize a set of national level data categorized by province/region and our interrupted time series study design allowed us to implement data that were objectively defined, easy to collect, simple to present graphically and straightforward to interpret.

Our study also had several weaknesses, the most important of which is that we were only able to determine correlations and associations between the onset of the COVID-19 pandemic and each of these other infectious disease pathogens/syndromes. Based on our study design, we were unable to ascertain causality. Moreover, several of these infectious disease pathogens/syndromes overlap to varying degrees with symptoms and manifestations of COVID-19 and could easily have led to misclassification bias during data collection. Due to efforts made early on by the Pakistani Government, especially under NCOC, to control COVID transmission using various non-pharmaceutical containment measures inclusive of lockdowns, implementation of masking, sanitization and social distancing policies and educational campaigns [17], there was likely an early reduction in the incidence of influenza and other respiratory infections [32] which could certainly have impacted our findings. Notably, however, many regions of the country have had poor access to masks and hand sanitizers, particularly the rural parts of the country [33], and the countrywide lockdowns were short-lived for financial reasons, ending in May 2020. 

There﻿ also may have been differences in data collection resources in various parts of the country at some time points, as well as national changes in data collection methodologies over time, as the Pakistani infectious disease surveillance system has evolved and somewhat improved over the past few years from completely paper-based to primarily electronic [34–36]. Nevertheless, despite some improvements in technology, it is notable that several infections that are preventable through appropriate surveillance and coupled public health efforts, including tuberculosis, meningitis, malaria, typhoid and hepatitis B and C, are still responsible for nearly half of the deaths in the country [37]. Overcoming major financial and logistic barriers, through collaboration with international partners, will be critical in developing a more vigorous and reliable national surveillance system—with laboratory integration—to properly serve the needs of the country in a sustainable fashion [38, 39]. As a result of the existing system, our surveillance data was, therefore, not consistently robust and comprehensive throughout the entire reporting interval, potentially resulting in imperfect capture of monthly incidence for some pathogens/syndromes. Fortunately, there were no missing data during the pandemic period and, thus, no need was ascertained to impute data. Importantly, however, the disease definitions we used, though based on Pakistani national surveillance protocols, are by their nature non-specific and somewhat difficult to compare with one another—some representing specific pathogens and others representing syndromes. Moreover, we worked on aggregated data and did not have access to case-based data; therefore, were unable to cater for confounder and effect modifiers at study design or analysis levels.

It is evident that the incidence, surveillance, and reporting of the studied diseases were affected considerably with progression of the pandemic. Whatever the situation was, it can be assumed that it resulted in adverse consequences with respect to assessing true infectious disease burden and capturing all outbreaks. As a result, many cases of these endemic infectious disease pathogens/syndromes have likely been present in Pakistan over the past 24 months, but not captured and mitigated by the health system, resulting in uncontained transmission to susceptible populations. Notably, there is a growing body of literature that corroborates the fact that several endemic infectious diseases in Pakistan have actually become more prevalent as a result of a diversion of scarce resources towards combatting the COVID-19 pandemic. Weakened and ineffective approaches towards the prevention and control of HIV disease, poliomyelitis, Dengue fever, tuberculosis and mucormycosis infections with a resulting increasing burden of disease have recently been well-documented and highlighted in Pakistan, particularly over the past couple years [40–44]. We believe that only time will demonstrate the full impact of COVID-19 on these and other endemic diseases, and the challenges that will need to be overcome just to achieve the same level of control as had been acquired prior to the pandemic. Ultimately, such a situation is likely to result in further outbreaks, hyper-endemic pathogens, or even other epidemics.

## Conclusions

Despite the presence of surveillance systems for several infectious disease pathogens/syndromes in Pakistan, eradication has been difficult, given the number of concurrent endemic pathogens, resource limitations, and the vastness of the population. The COVID-19 pandemic has affected surveillance of other important ongoing endemic infectious diseases, including AURI, viral hepatitis, pneumonia, diarrhea, typhoid fever, measles, NNT, and malaria. Though the exact reasons for this cannot be determined by our study, we confidently postulate that this was in some degree due to a lack of sufficient resources, and diversion of existing resources towards addressing the COVID-19 pandemic. In particular, there is a lack of laboratory diagnostic capability to differentiate between COVID-19 and several other infectious disease pathogens endemic to Pakistan [16].

It is recommended that surveillance and reporting systems of diseases should be strengthened and coordinated so that any change in disease trends may be detected and responded to at the earliest opportunity to contain disease spread locally. There is also a need to develop public health emergency preparedness plans to cater to outbreaks and health emergencies more efficiently and effectively. Countrywide awareness and risk communication regarding personal protective measures, access to safe food and water supply, and education and access to vaccination are among interventions that are desperately needed in Pakistan’s battle against these various infectious diseases. It was recognized early on in the COVID-19 pandemic that vaccination would offer the global community the best chance at controlling it. Though many countries were early adopters [45], Pakistan has suffered from the twin perilous ideologies of vaccine hesitancy [46] and vaccine nationalism [47, 48]. The former has been a longstanding problem in the country and is the major reason for which it has been one of the last bastions of poliomyelitis in the world in the years leading up to the pandemic, whereas the latter has been deleterious not just for low and middle income countries such as Pakistan but for the developed world as well (despite each nation believing that it is acting in its best self-interest) [47, 48], resulting in the development of new variant strains and lengthening the duration of the pandemic. Regrettably, if these factors persist, the consequences will be much worse for Pakistan's dilapidated healthcare sector, which is in dire need of development and implementation of concrete and achievable policies for the country’s largest-ever mass vaccination campaign to achieve set targets within planned timelines. In addition, educational campaigns must be carried out to address vaccine misconception and hesitancy issues through various local and mass-media resources such as television, radio, newspapers, websites and social media [47]. If we delay or fail to do this appropriately, then control of the pandemic for Pakistan could become an unfulfilled dream, and more precious lives will be lost.

Despite some limitations, our study was a starting point for characterizing impacts of the COVID-19 pandemic on other endemic infectious diseases in Pakistan as well as in other developing countries. We are convinced that such work will be pivotal in making the case that more resources are required to ensure that we do not lose sight of other control and eradication efforts, while our attention is captured by this all-consuming pandemic. If we are to act otherwise, many of the great efforts that have been made over the previous decades may be consigned to oblivion.

## Supplementary Information

**Additional file 1: Figure S1.** Boxplots of distributions of monthly reported cases of various diseases pre-COVID and after the onset of the COVID-19 pandemic in five provinces/regions of Pakistan.

## Data Availability

The datasets generated and/or analyzed during the current study are not publicly available and were acquired with permission from the Government of Pakistan MoNHSR&C DHIS database. These data will not be provided as a supplement to this manuscript but may be made available from the corresponding author on reasonable request.

## Abbreviations

CCHF: Crimean-Congo Hemorrhagic fever
COVID-19: Novel coronavirus 2019
SARS-CoV-2: Severe acute respiratory syndrome coronavirus 2
NCOC: National Command and Operations Center
MoNHSR&C: Ministry of National Health Services Regulations and Coordination
KPK: Khyber Pakhtunkhwa
AJK: Azad Jammu & Kashmir
GB: Gilgit Baltistan
IDSR: Integrated Disease Surveillance Response System
AURI: Acute upper respiratory infection
NNT: Neonatal tetanus
DHIS: Demographic Health Information System
IQR: Interquartile range
HAC: Heteroskedasticity and autocorrelation-consistent
IRRs: Incidence rate ratios
CIs: Confidence intervals
OPDs: Outpatient treatment departments
URTI: Upper respiratory tract infection
