# Supplementary figures and images for: Associations of the COVID-19 pandemic with the reported incidence of important endemic infectious disease agents and syndromes in Pakistan
Source: BMC Infect Dis. 2022 Nov 26;22:887. doi: 10.1186/s12879-022-07869-3 (PMC9701436; doi:10.1186/s12879-022-07869-3)

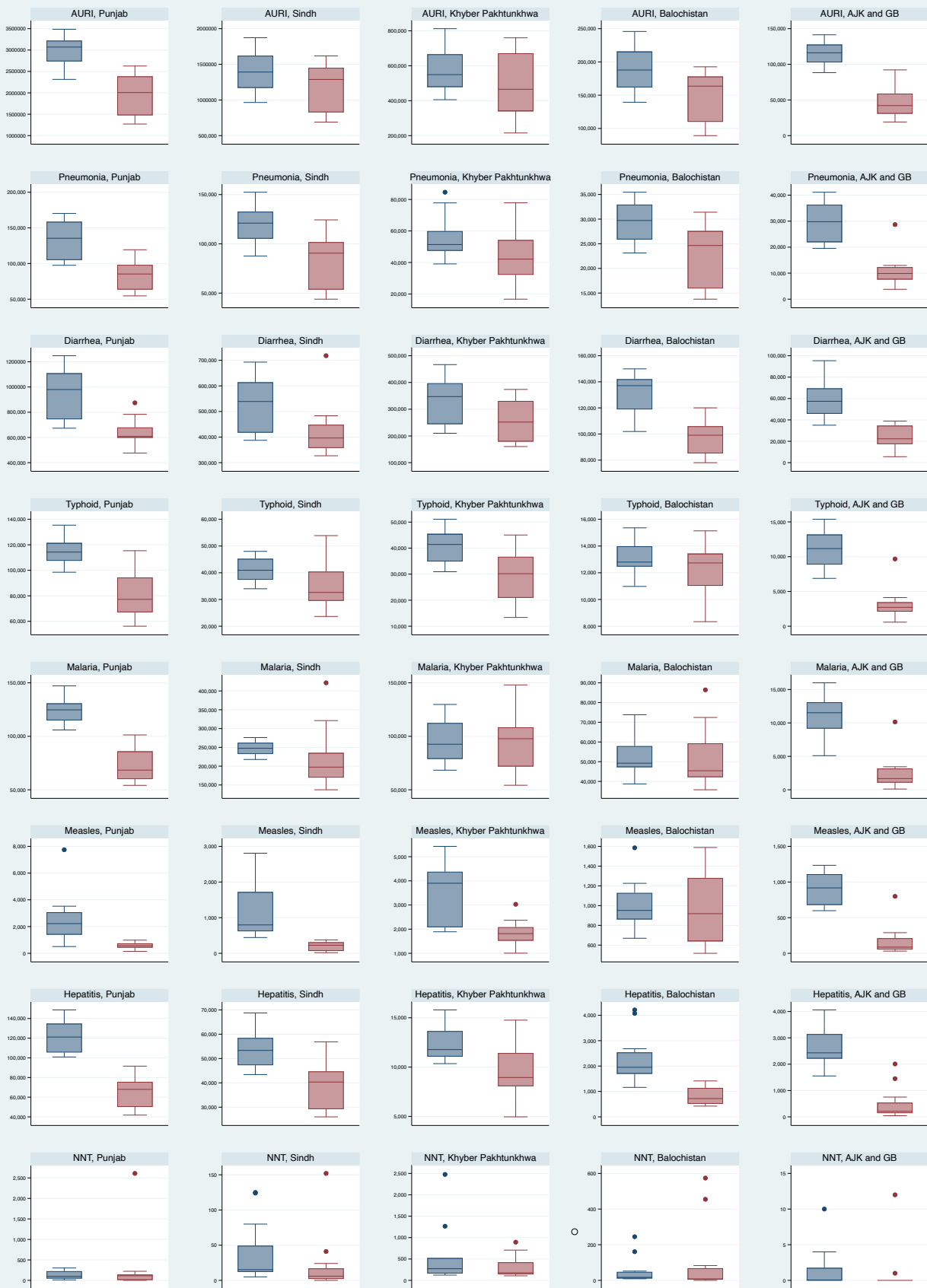

Pre-COVID Period (2018-20)

COVID Period (2020-21)

Supplement: Supplementary file 1 — Additional file 1: Figure S1. Boxplots of distributions of monthly reported cases of various diseases pre-COVID and after the onset of the COVID-19 pandemic in five provinces/regions of Pakistan. [file 12879_2022_7869_MOESM1_ESM.pdf]
